# Formation of the Secondary Abscission Zone Induced by the Interaction of Methyl Jasmonate and Auxin in *Bryophyllum calycinum*: Relevance to Auxin Status and Histology

**DOI:** 10.3390/ijms21082784

**Published:** 2020-04-16

**Authors:** Agnieszka Marasek-Ciolakowska, Marian Saniewski, Michał Dziurka, Urszula Kowalska, Justyna Góraj-Koniarska, Junichi Ueda, Kensuke Miyamoto

**Affiliations:** 1 Research Institute of Horticulture, Konstytucji 3 Maja 1/3, 96-100 Skierniewice, Poland; marian.saniewski@inhort.pl (M.S.); urszula.kowalska@inhort.pl (U.K.); justyna.goraj@inhort.pl (J.G.-K.); 2 The Franciszek Górski Institute of Plant Physiology, Polish Academy of Sciences, Niezapominajek 21, 30-239 Kraków, Poland; michal.dziurka@gmail.com; 3 Department of Biological Science, Graduate School of Science, Osaka Prefecture University, 1-1 Gakuen-cho, Naka-ku, Sakai, Osaka 599-8531, Japan; ueda@b.s.osakafu-u.ac.jp; 4 Faculty of Liberal Arts and Science, Osaka Prefecture University, 1-1 Gakuen-cho, Naka-ku, Sakai, Osaka 599-8531, Japan

**Keywords:** auxin, abscission, *Bryophyllum calycinum*, histology, hormonal crosstalk, methyl jasmonate, stem

## Abstract

The interaction of methyl jasmonate (JA-Me) and indole-3-acetic acid (IAA) to induce the formation of the secondary abscission zone in the middle of internode segments of *Bryophyllum calycinum* was investigated in relation to auxin status and histology. When IAA at 0.1% (*w*/*w*, in lanolin) was applied to the segments, the formation of the secondary abscission zone at a few mm above the treatment in the apical direction was observed. On the contrary, IAA at 0.5% (*w*/*w*, in lanolin) did not induce the formation of the secondary abscission zone. JA-Me at 0.5% (*w*/*w*, in lanolin) applied to the middle of internode segments kept in the normal (natural) or inverted positions also induced the formation of the secondary abscission zone below and above parts of the treatment. IAA at 0.5% applied to the cut surface of the upper part of the segments completely prevented the formation of the secondary abscission zone induced by JA-Me. Simultaneous application of IAA 0.5% with JA-Me 0.5% in the middle part of the internode segments induced the formation of the secondary abscission zone at 10 mm to 12 mm above the treatment. Histological analyses indicated that the formation of the secondary abscission zone was characterized by the presence of newly synthesized cell plates that resulted from periclinal cell division within one layer of mother cells in stems. The effects of IAA (0.1%) and JA-Me (0.5%) on the formation of the secondary abscission zone were histologically similar. Comprehensive analyses of plant hormones revealed that the balance of the endogenous levels of IAA in both sides adjacent to the abscission zone was significantly disturbed when the secondary abscission formation was induced by the application of IAA. These results strongly suggest that an auxin gradient is important in the formation of the secondary abscission zone in the internode segments of *B. calycinum*, and IAA gradient results from polar IAA transport from the application site. IAA is important in the regulation of formation of the secondary abscission zone induced by JA-Me. Further possible mechanisms of the formation of the secondary abscission zone in the internode segments of *B. calycinum* are also discussed in the interaction of JA-Me and IAA.

## 1. Introduction

Abscission is commonly associated with the sequence of a regulated process resulting in natural shedding (separation) of plant organs such as leaves, branches, flowers and fruits, from the parent plant [1,2,3,4,5,6]. During abscission, mechanical weakening of cell walls at the abscission zone is brought about by the degradation of the middle lamella by multiple cell-wall degrading enzymes such as cellulase, polygalacturonases, pectin methyl esterases, etc. [3,7,8,9].

A decrease in auxin levels is considered to provide the first signal for abscission [10]. Meir et al. [11] suggested that stress-induced auxin depletion plays an important role in abscission. On the other hand, methyl jasmonate (JA-Me) is also well known as an inducer of abscission in different organs in plants [12,13,14,15,16,17,18].

In most cases, the site and the time of abscission zone formation is genetically determined in each organ, and normally they do not differentiate further once formed. Webster and Leopold [19] have found that one or multiple abscission zones formed spontaneously in the internode of the stem explants of *Phaseolus vulgaris,* and designated them as secondary abscission zone formations. The secondary abscission is formed in tissues away from a recognizable abscission zone, in positions that are not defined in the intact plants. Anatomical changes precluding separation in internodal and nodal regions are similar to those occurring during leaf abscission [19]. The induction of secondary abscission is possible especially in in vitro systems of various plants. [4,20,21,22,23,24,25,26,27,28]. 

The secondary abscission zone has been considered to be formed by some signals possessing a specific functional competence between neighboring cells [29,30,31]. However, studies on the secondary abscission zone formation are very limited in comparison to those on primary abscission zones. Crassulaceae including *Bryophyllum calycinum*, *Kalanchoe blossfeldiana* and *Crassula lycopodioides* is one of the important plant families for horticulture. Methyl jasmonate (JA-Me) has been demonstrated to induce the secondary abscission zone formation and senescence in several types of stem explants of *Bryophyllum calycinum*, such as the internode segment only, and the internode segment with nodes and without leaves, when it was applied on the stem as a lanolin paste [32]. It should be mentioned that in the presence of small leaves in stem segments, JA-Me induced the secondary abscission zone formation and senescence, but the presence of larger leaves completely inhibited such effects of JA-Me. Auxin, indole-3-acetic acid (IAA), applied to a detached leaf totally prevented the formation of the secondary abscission zone in the stem tissues induced by JA-Me [32], suggesting that the interaction of JA-Me with IAA transported from leaves plays an important role in the secondary abscission zone formation. Only IAA application is also found to substantially induce the formation of the secondary abscission zone in internode explants, petiole segments, the petiole after excision of leaf blade in intact plants, and decapitated stems in intact plants of *B. calycinum* [33]. Therefore, it is worthwhile to study the dual physiological effects of auxin, especially interacting with JA-Me, on the formation of the secondary abscission zones in stem segments of *B. calycinum* in the aspects of histochemical and biochemical investigations. 

The purpose of this study is to histologically compare the formation of the secondary abscission zones induced by JA-Me and IAA, and to propose the mechanisms of the JA-Me and IAA interaction based on the gradient of endogenous IAA levels in the formation of the secondary abscission zones in stem segments of *B. calycinum*. 

## 2. Results 

### 2.1. Effect of IAA or JA-Me on the Formation of the Secondary Abscission Zone in the Internode Segments of Bryophyllum calycinum

**Figure 1:**
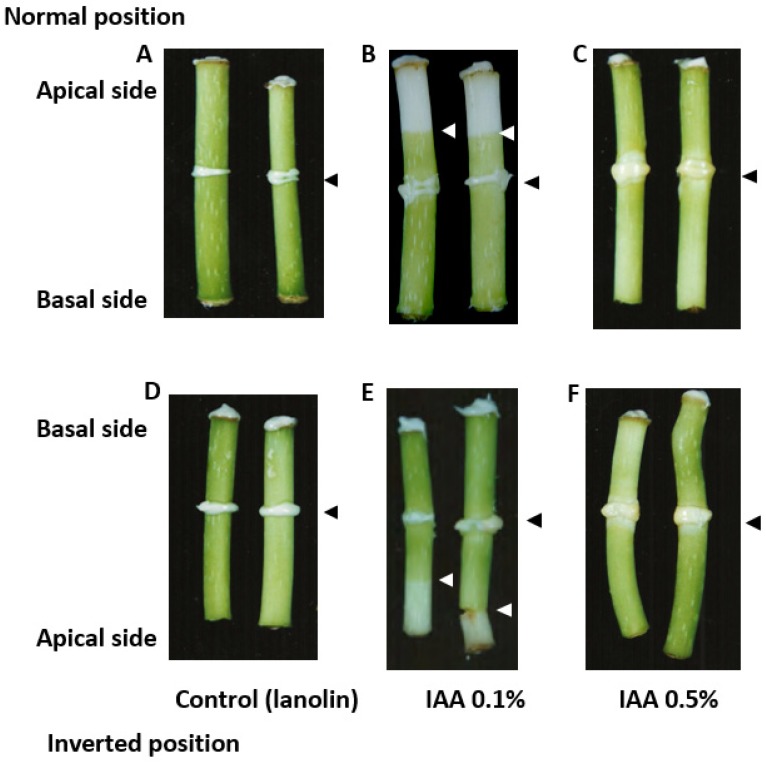
The effect of indole-3-acetic acid (IAA) at concentrations of 0.1% and 0.5% on the formation of the secondary abscission zone in the internode segments of *B. calycinum*. (**A**,**D**): lanolin (control) applied only to the middle of the segments in the normal position (**A**) and inverted position (**D**); (**B**,**E**): IAA at 0.1% was applied to the middle of the segments in the normal position (**B**) and inverted position (**E**); (**C**,**F**): IAA at 0.5% was applied to the middle of the segments in the normal (**C**) and inverted position (**F**). The segments were incubated under light conditions and photographed 9 days after the treatment. Black arrowheads indicate the place of treatment, and white arrowheads indicate the place of the formation of the secondary abscission zone.

A concentration of 0.1% was applied to the middle of internode segments without nodes of *B. calycinum,* and the segments were then kept in the normal (natural) position under light conditions for 9 days. The formation of the secondary abscission zone was found a few mm above the treatment area in the apical or acropetal direction, whereas lanoline applied on its own had no effect, as shown in Figure 1A,B. Senescence or the loss of chlorophylls in the acropetal direction of the internode segments above the secondary abscission zone was induced by the application of IAA at 0.1%, even when they were kept in the inverted position, as shown in Figure 1E.

The formation of the secondary abscission zone with chlorophyll disappearance is suggested to be independent of the orientation of the internode segments (basal end down or basal end up) when IAA at 0.1% was applied to the middle of the segments. Similar effects of IAA at 0.1% on the formation of the secondary abscission zone were obtained in the internode segments with lower nodes and on decapitated shoots of growing *B. calycinum*, as displayed in Figure S1.

In contrast, IAA at a concentration of 0.5% did not induce the formation of the secondary abscission zone in the internode segments of *B. calycinum* regardless of the keeping position of the segment, when it was applied in the middle of the internode segments, as shown in Figure 1C,F. These results indicate that IAA-induced the formation of the secondary abscission zone is dependent on the concentration of IAA applied. 

### 2.2. Histological Analyses of the Formation of the Secondary Abscission Zone Induced by IAA or JA-Me in the Internode Segments of B. calycinum 

As already demonstrated [32], JA-Me alone at a concentration of 0.5% in lanolin paste applied to the middle of internode segments of *B. calycinum* substantially induced the formation of the secondary abscission zone. To compare the processes of the formation of the secondary abscission zone induced by IAA and JA-Me, histological observations were carried out. Stem pieces of 0.7 cm in the middle of internodes were cut from 1 cm above the point of the application of lanolin paste of IAA at 0.1% or JA-Me at 0.5% on the sixth, eighth and ninth day after the treatment. 

**Figure 2:**
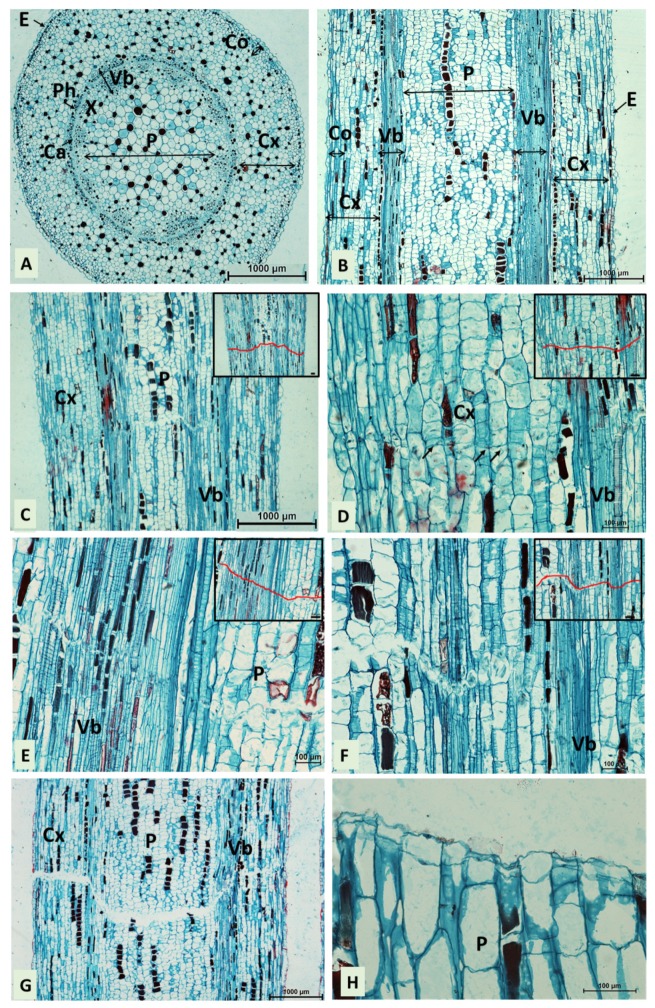
Microscopy investigation of the secondary abscission zone in the middle of stem internodes induced by IAA 0.1% (longitudinal sections). (**A**,**B**) Anatomical details of the stem in the middle of internodes not treated with hormones (control) stained with safranin-fast green. (**A**): a cross-section; (**B**): a longitudinal section; CA–cambium; Cx–cortex; E–epidermis; CO–collenchyma; P–pith; PH–phloem; VB–vascular tissues; X–xylem. (**C**) the beginning of the formation of an abscission zone, cell separation visible in pith, photographed on the eighth day after treatments; (**D**) the formation of an abscission zone in the cortex; (**E**) cell separation in the cortex and pith; (**F**) the formation of an abscission zone in the vascular tissue region; (**G**) cell separation visible in all tissues of the stem internode; (**H**) the secondary abscission completed, photographed on the ninth day after treatments; (**C**–**F**) the insert displays the outline of the creation of abscission zones (red); bars in the inserts represent 100 µm; Cx–cortex; N–nucleus; P–pith; VB–vascular tissues. Arrows denote cell-plate forming between daughter cells.

The transverse and longitudinal sections of the internode of *B. calycinum* treated with lanolin only (control) are shown in Figure 2A,B. The outer surface of the stem is covered by dermal tissue comprising of epidermis with pronounced cuticles, and a few layers of collenchyma cells. Further into the stem, the cortex parenchyma comprising of large, vacuolated cells is observed, and from inside it adheres to a ring of vascular tissue with xylem and phloem separated by a cambial zone. The central part of the stem (pith) is occupied by a core parenchyma with round, vacuolated cells, increasingly larger towards the center. 

The first anatomical symptoms of the formation of the secondary abscission zone in the middle of the internode segments treated with IAA at 0.1% were observed on the eighth day of the treatment, as shown in Figure 2C. The region of separation is comprised by many cell types, e.g., parenchyma cells of the pith and cortex, and cells of vascular tissues. Initially, cell wall loosening occurred in the central parenchymatous region of internode (pith parenchyma) at a distal green and a proximal yellow junction of cells, as displayed in Figure 2C. No increase in cell size in any region of the stem internode was observed prior to separation. In the cortex, cell wall forming between daughter cells after cell divisions was observed, shown in Figure 2D. In contrast, no divisions were observed in vascular bundles and epidermal cells which are mechanically ruptured apart, as displayed in Figure 2E,F. The secondary abscission was completed on the ninth days after IAA treatment. After the separation, the green side was completed with a single periderm-like layer which is comprised of smaller and densely packed cells when compared to the adjacent cells of the cortex and pith as shown in Figure 2G,H. 

**Figure 3:**
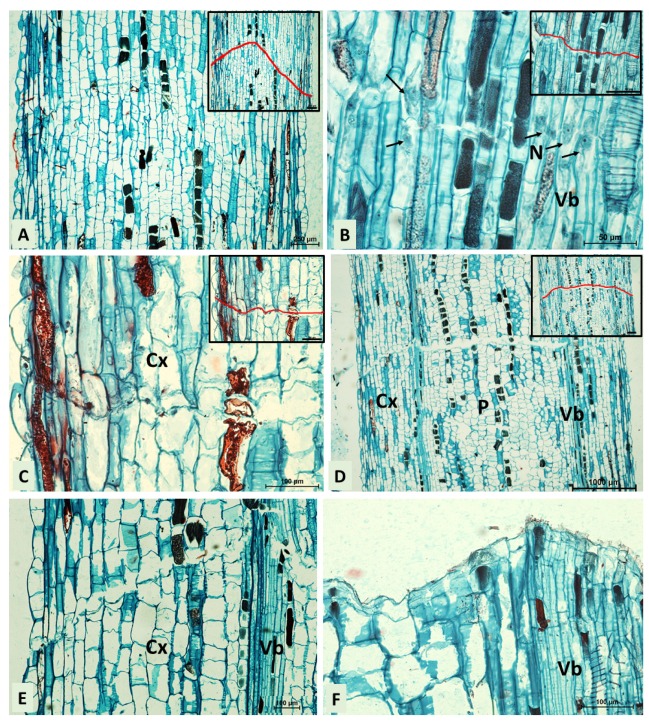
Microscopy investigation of the secondary abscission zone in the middle of stem internodes induced by methyl jasmonate (JA-Me) 0.5% (longitudinal sections). (**A**) the beginning of the formation of an abscission zone that forms between yellow and green tissues of the internode; (**B**) the formation of an abscission zone in the vascular tissue region. Note the nuclei lying in close proximity to the line of separation (arrow); (**C**) the formation of an abscission zones in the cortex; (**A**–**C**) photographed on the eighth day after treatments; (**D**) cell separation visible in all tissues of the stem internode, photographed nine days after treatments; (**E**) an enlargement of Figure 3D, cell separation in the cortex and vascular tissues; (**F**) the secondary abscission completed, photographed on the ninth day after treatments; (**A**–**D**) the insert displays the outline of the creation of abscission zones (red); bars in the inserts represent 100 µm; Cx–cortex; N–nucleus; P–pith; VB–vascular tissues. Arrows denote cell-plate forming between daughter cells.

Figure 3 shows anatomical changes accompanying the secondary abscission zone formation after applying JA-Me at 0.5% to the middle of internodes. The abscission zone was distinguished on the eighth day, and it was characterized by the presence of newly synthesized cell plates resulting from periclinal cell division within one layer of mother cells, as shown in Figure 3A–C. In the cortex and around the vascular tissue, the aggregation of nuclei in close proximity to the newly synthesized cell walls was observed, as displayed in Figure 3B,C. The separation process was initiated in the pith, which is comprised of large, loosely laid parenchyma cells as shown in Figure 3D, and then spread to the vascular tissue and the cortex presented in Figure 3E. The secondary abscission was completed on the ninth day after JA-Me application. The secondary abscission zone is consisted of a single protective layer of cells resembling periderm, shown in Figure 3F.

### 2.3. Changes in the Levels of Endogenous Plant Hormones in Relation to the Formation of the Secondary Abscission Zone Induced by IAA

**Figure 4:**
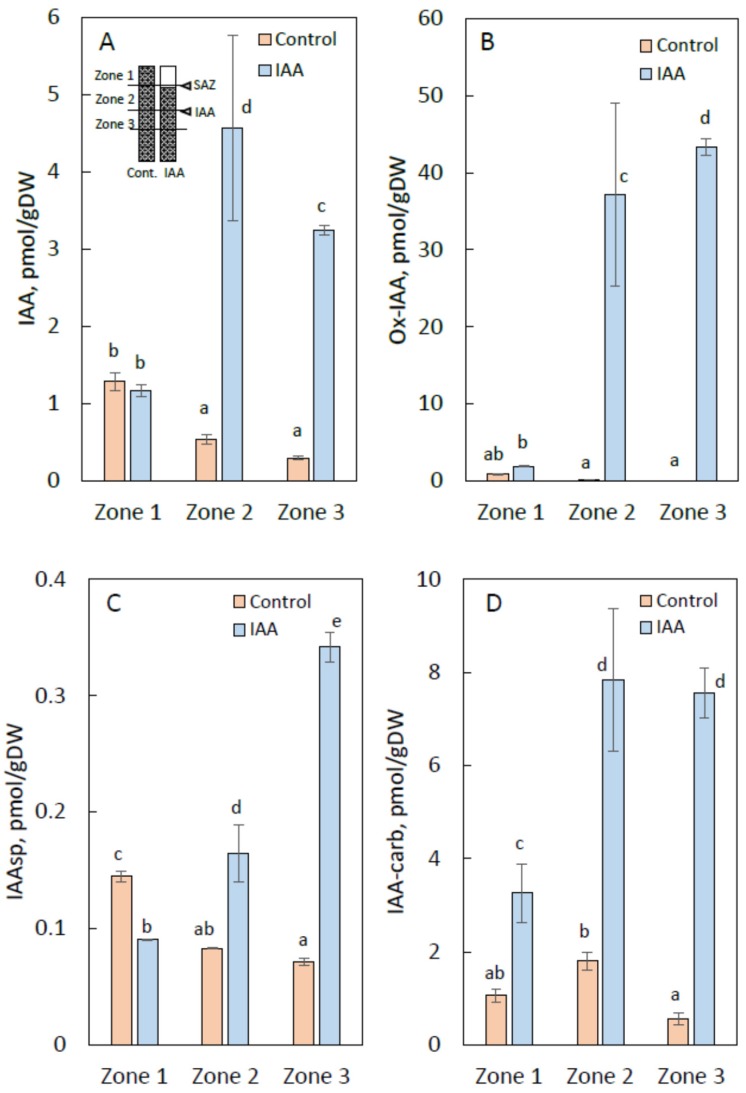
The effect of IAA at a concentration of 0.1% applied to the middle of the internode segments of *B. calycinum* at endogenous levels of IAA (**A**), Ox-IAA (**B**), IAAsp (**C**) and IAA-carb (**D**). Stem segments shown in Figure 1A,B were used for plant hormone analyses. The following samples of the stem were taken for analysis after the treatment with IAA: the top yellowing part at the early stage of the secondary abscission formation (zone 1), the green part above the treatment (zone 2), and green part of the internode below the treatment (zone 3). Control samples were taken from equivalent zones in the segments treated with lanolin only. Values are the mean ± standard error (*n* = 3). Different letters indicate statistic difference by Duncan’s multiple range test, with *p* < 0.05 after ANOVA.

As shown in Figure 1, when IAA at 0.1% was applied to the middle of the internode of *B. calycinum,* the secondary abscission zone was found a few mm above the treatment in the apical direction. The status of the endogenous plant hormones in relation to the induction of the secondary abscission zone was determined. Zone 1, zone 2 and zone 3 in the internode segments were harvested for plant hormone analyses (see the inserted figure in Figure 4A). Comparable samples were taken from the control segments treated with lanolin only. 

The following auxins and its related compounds were successfully identified in the stem segments of *B. calycinum*: IAA, indole-3-acetyl-l-aspartic acid (IAAsp), indole-3-acetyl-4-glutamic acid (IAA-Glut), indole-3-acetic acid methyl ester (IAA-Me), indole-3-carboxylic acid (IAA-carb), oxindole-3-acetic acid (Ox-IAA), 4-chloroindole-3-acetic acid (4-Cl-IAA), 5-chloroindole-3-acetic acid (5-Cl-IAA) and indole-3-butyric acid (IBA), shown in Table S1. In the control segments, endogenous levels of IAA gradually decreased from zone 1 to zone 3, whereas no differences were observed between zone 2 and zone 3. On the contrary, endogenous levels of IAA in zone 2 in IAA-treated stem segments were extremely high compared to those in zone 1 and zone 3. In this case, endogenous levels of IAA in zone 3 were higher than those of zone 1, indicating that IAA treated as a lanolin paste moved in the basipetal direction rather than the acropetal direction, as shown in Figure 4A. 

The endogenous levels of Ox-IAA, a major metabolite of IAA, in zone 2 and zone 3 of IAA-treated stem segments were also extremely high, although very low levels were recorded in the control stem segments, as displayed in Figure 4B. As shown in Figure 4C,D, endogenous levels of IAAsp and IAA-carb in zones 1 to 3 were almost same, with a gradual increase from zone 1 to zone 3. Endogenous levels of IAA-carb were about ten times higher than those of IAAsp. The reason for these results has not yet been clarified, but there are two possible explanations. One is that exogenously applied IAA moved basipetally and then metabolized into OxIAA, IAAsp and IAA-carb. Another is that exogenously applied IAA immediately metabolized into these metabolites and then these compounds moved basipetally rather than acropetally and similarly to IAA. IAA exogenously applied to the stem segments was also metabolized into IAA-Glut, 4-Cl-IAA, 5-Cl-IAA, IAA-Me, and IBA. Endogenous levels of these metabolites were almost similar and had relatively small differences when compared to the control stem segments shown in Table S1. 

**Figure 5:**
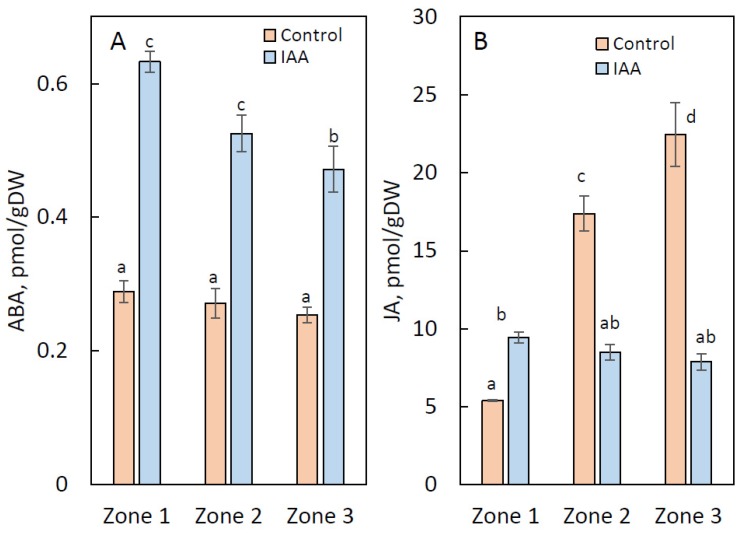
The effect of IAA at a concentration of 0.1% applied to the middle of the internode segments of *B. calycinum* at endogenous levels of ABA (**A**) and JA (**B**). Others are the same as in Figure 4.

The endogenous levels of abscisic acid (ABA) in zones 1 to 3 of IAA-treated stem segments were about twice higher than those in control segments, shown in Figure 5A. Endogenous levels of jasmonic acid (JA) in zones 2 and 3 of IAA-treated stem segments were significantly low compared with the control segments, where endogenous levels of JA gradually increased from zone 1 to zone 3, as displayed in Figure 5B. On the other hand, levels of JA-Me and 12-oxo-phytodienoic acid (OPDA) in the IAA-treated stem segments were almost the same as those of the control segments, and the endogenous levels of these compounds in the control and IAA-treated stem segments were extremely low compared to those of JA, as shown in Table S2. Salicylic acid (SA) and benzoic acid (BA) contents were almost similar both in the IAA-treated and control stem segments, also displayed in Table S2. 

As shown in Table S3, the contents of all identified cytokinins, trans-zeatin (t-Z), cis-zeatin (c-Z), trans-zeatin riboside (t-ZR), cis-zeatin riboside (c-ZR), isopentenyladenine (IP), isopentenyladenosine (IPAD), kinetin (KIN) and kinetin riboside (KIN-R) in the IAA-treated stem segments were almost the same as those of the control segments. Gibberellin 1 (GA_1_), GA_3_, GA_4_, GA_5_, GA_6_, GA_7_, GA_9_ and GA_20_ were identified in the stem segments, and similar levels of these gibberellins were found in both the IAA-treated and control stem segments, as shown in Table S4. These results strongly suggest that cytokinins and gibberellins do not contribute to the formation of the IAA-induced secondary abscission zone in *B. calycinum*.

### 2.4. The Effects of the Interaction between JA-Me and IAA on the Formation of the Secondary Abscission Zone in the Internode Segments with or without the Node of B. calycinum

**Figure 6:**
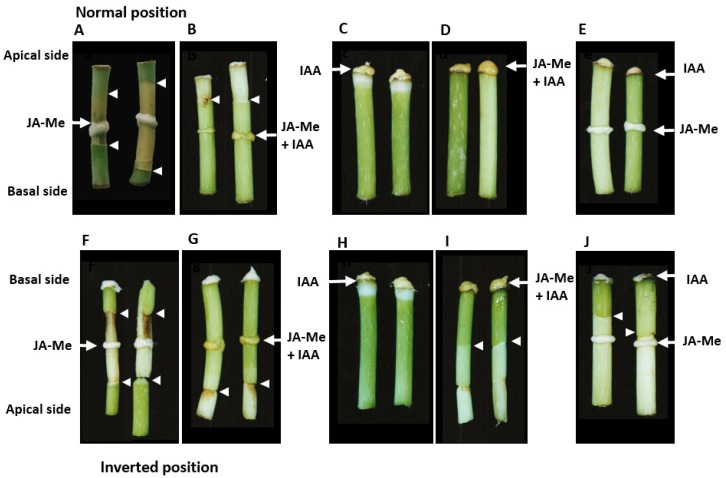
The effect of IAA 0.5%, JA-Me 0.5% and their mixture on the formation of the secondary abscission zone depending on the place of treatment in the excised segments of the internode of *B. calycinum*. After the treatment, the internode segments were kept in the normal position (**A**–**E**) and the inverted position (**F**–**J**), and photographed ten days after treatments; (**A**,**F**) JA-Me applied on its own to the middle of the internode and lanolin to the top; (**B**,**G**) IAA applied together with JA-Me to the middle of internode and lanolin to the top; (**C**,**H**) IAA alone applied to the top of internode; (**D**,**I**) JA-Me and IAA applied together to the top; (**E**,**J**) JA-Me applied to the middle and IAA applied to the top of the segment. White arrows indicate the place of treatment, and white arrowheads indicate the place of the formation of the secondary abscission zone.

As shown in Figure 6, JA-Me alone at a concentration of 0.5% in lanolin paste applied to the middle of the internode segments kept in the normal and inverted positions substantially induced the formation of the secondary abscission zone below and above the treatment, as shown in Figure 6A,F. No necrosis was substantially observed in the present study. This observation strongly suggests that the effect of JA-Me is hormonal but not nonspecific or toxic.

When a mixture of IAA 0.5% + JA-Me 0.5% was applied to the middle of the internode segments without the node, the secondary abscission zone was formed about 10 to 12 mm above the treatment in the apical or the acropetal direction, as shown in Figure 6B,G. 

Interestingly, IAA at 0.5% applied alone to the top of an internode segment without the node caused bleaching and the loss of chlorophyll just below the place of application, but did not cause the formation of the secondary abscission zone, regardless of the application of IAA in the normal and inverted positions, as shown in Figure 6C,H. A Mixture of IAA 0.5% + JA-Me 0.5% applied to the top of internode segments kept in the normal position did not cause the formation of the secondary abscission zone, but substantially induced it in internode segments kept in the inverted position, as shown in Figure 6D,I. In contrast, the application of JA-Me 0.5% to the top of internode segments kept in the natural position induced abscission zone formation and senescence in the upper half of the segments as documented earlier [32]. 

The application of JA-Me at 0.5% to the middle of the segments substantially induced secondary abscission zones above and below the treatment as shown in Figure 6A,F. However, the application of IAA to the acropetal or the apical sides completely inhibited the formation induced by the application of JA-Me at 0.5% to the middle and in the top of the segment, as displayed in Figure 6D,E. When IAA at 0.5% was applied to the basipetal or basal sides of the segments, the formation of the secondary abscission zone induced by JA-Me applied to the middle of the segments was observed. These facts strongly suggest that the effect of JA-Me to induce the formation of a second abscission zone was interfered by that of basipetally transported IAA. 

In the internode segments with nodes, the effects of IAA and JA-Me applied to the middle or the cut surface of the segments kept in the normal and inverted positions were almost same as those in the segments without nodes shown in Figure 6 (Figure S2). 

## 3. Discussion

### 3.1. The Mode of Action of IAA to Induce the Formation of the Secondary Abscission Zone in the Stem of B. calycinum 

When IAA at 0.1% was applied to the middle of the internode segments of *B. calycinum* kept in normal and inverted positions, the formation of the secondary abscission zone a few mm above the treatment in the acropetal direction was observed, as shown in Figure 1B,E. In contrast, IAA at 0.5% alone did not induce the formation of the secondary abscission zone in the internode segments of *B. calycinum* when it was applied in the middle of the internodes, as displayed in Figure 1C,F. The reasons for the different effects of IAA depending on its concentrations have not yet been clarified, but it might be a possible explanation might be that an appropriate IAA concentration in the segments is responsible for inducing the formation of the secondary abscission zone since IAA is only transported basipetally. 

As shown in Figure 4A and Table S1, physicochemical analyses revealed relatively low levels of IAA in the yellowing top part of the stem segments (above the abscission zone formation) in IAA-treated segments in comparison to the lower parts just above and below the treatments. This result strongly supports the hypothetical mechanism of IAA to induce the formation of the secondary abscission zone as mentioned above. Based on these results, it is possible to mention that auxin gradient in the segments is an important factor in the induction of the secondary abscission zone. Ito and Nakano [10] reported that a decrease in auxin levels is considered to provide the first signal for the secondary abscission zone formation regarding the pedicel abscission zone formation in tomatoes.

Similar to IAA, relatively low levels of Ox-IAA in the yellowing top part of stem segments (above the abscission zone formation) in IAA-treated segments were recorded in comparison to the lower part directly above and below the treatments, as shown in Figure 4B. The levels of Ox-IAA were extremely high compared with those of IAA. Similar results have been reported showing a huge accumulation of Ox-IAA in the case of Arabidopsis plants [34]. The oxidation of exogenously applied IAA to Ox-IAA has also been demonstrated to be the major catabolic pathway for IAA in maize endosperm, whereas Ox-IAA is a naturally occurring inactive compound in shoot and endosperm tissues of maize [35,36]. The rate of oxidation of IAA to Ox-IAA could play a role in the regulation of endogenous levels of IAA, resulting in the IAA-induced formation of the secondary abscission zone in the segment kept in the normal position. 

Auxin conjugates are thought to play an important role as storage forms for the active IAA [37]. The accumulation of IAA metabolites is also considered to be one of the mechanisms that regulates auxin homeostasis and auxin response [38,39,40]. The endogenous status of IAAsp and IAA-carb were quite similar to that of IAA after the application of IAA at 0.1% to the middle of the segment as shown in Figure 4C,D, suggesting that IAA metabolic processes are also important for hormonal balance to regulate IAA-induced growth and development.

ABA and JA are well-known to induce abscission and senescence [13,14,41]. The application of IAA to the middle of the segment substantially increased ABA levels in the yellowing top part of the stem segments (above the abscission zone formation), and in the lower part directly above and below the treatment zones in the IAA-treated segments, with endogenous levels of ABA being higher in the yellowing top part of the stem segments, as shown in Figure 5A. On the other hand, IAA treatment inducing the secondary abscission zone could not increase the endogenous levels of JA as shown in Figure 5B, and had little effect on the endogenous levels of JA-Me and OPDA in all parts of the stem segments, as shown in Table S2. These facts suggest that exogenously applied IAA substantially regulates the formation of the secondary abscission zone via affecting the synthesis and metabolism of ABA. 

JA-Me treatment of stem segments may have induced the formation of the secondary abscission zone due to a decrease in IAA levels, since IAA totally prevented the induction of the secondary abscission zone in the stem segments of *B. calycinum* induced by JA-Me, as shown in Figure 6 and Figure S2.

An auxin gradient spanning the abscission zone has already been proposed to regulate the timing of organ separation [42]. In addition, Jin et al. [43] suggested that the formation of an abscission zone and the regulation of auxin transport in petioles of leaves in *Populus* kept in the dark were independent of ethylene signaling. Auxin might act in parallel, and independently of ethylene in hydrolysis of the middle lamellae. Tucker and Kim [5] concluded that auxin seems to be important in primary and secondary abscission systems, but understanding how cells might sense an auxin gradient is still unclear. 

### 3.2. The Effects of the Interaction between JA-Me and IAA on the Formation of the Secondary AbscissionZone in the Stem Segments of B. calycinum 

Our present experiments fully confirm our previous findings that JA-Me alone at a concentration of 0.5% in lanolin paste applied to the middle of internode segments substantially induced the formation of the secondary abscission zone [32]. In the internode segments of *B. calycinum*, only JA-Me at 0.5% applied to the middle of the internode segments kept in the normal and inverted positions substantially induced the formation of the secondary abscission zone below and above the treatment, as shown in Figure 6A,F. However, in the case of the segments treatment in the middle with a mixture of IAA 0.5% + JA-Me 0.5%, IAA prevented the formation of the secondary abscission zone below the treatment in stem segments with nodes. In stem segments without nodes, auxin prevented the formation of the secondary abscission zone below treatment, but induced it 10–15 mm above the treatment zone. It is possible that IAA applied to the middle of the segments is not only basipetally transported, but is also transported acropetally. Short distances may be travelled via the passive diffusion stream with it being loaded to appropriate transport systems, and finally it is transported back basipetally. 

It seems that abscission zone formation above the treatment with IAA 0.5% + JA-Me 0.5% in the middle of the internode is induced by JA-Me, and auxin protected senescence of the stem segments induced by JA-Me. The abscission zone is formed in the place where IAA is transported basipetally and it is still unknown whether acropetal auxin movement occurs only merely by diffusion or whether its rate is metabolically regulated. Warren Wilson P.M. et al. [22] and Warren Wilson J. et al. [23,24,25], on the basis of experiments with internodal explants of *Impatiens sultani*, presented hypothesis that the site of the secondary abscission is formed where the concentration of diffusive and polar transported auxin decreases locally in the apical direction. It should be mentioned here that auxin concentration is still higher in nodes than in internodes of *Impatiens sultani* [25]. 

JA-Me substantially induces the formation of the secondary abscission zone in the stem segments of *B. calycinum,* but the mechanism of the process is still unknown. IAA totally inhibited secondary abscission zone induction by JA-Me when they were simultaneously applied, indicating that the process induced by JA-Me is related someway to IAA. JA-Me was capable of inducing the secondary abscission zone above and below the treatments in the middle of stem segments of *B. calycinum*. This means that JA-Me is transported both in the acropetal and the basipetal directions in the stem segments. It has been reported that JA and JA-Me possess transportable properties throughout the plant [44,45,46]. It seems that the place of the secondary abscission zone in the stem segments above and below of treatment is decided by the distance of JA-Me transport in the stem, and JA-Me is responsible for IAA status and the change in concentrations of JA-Me and IAA. 

IAA and JA-Me applied alone in the middle of the internode segments induce the secondary abscission zone in the acropetal and the basipetal directions, suggesting that these compounds also directionally induce the secondary abscission zone. In the present experiments, we found that when a mixture of IAA 0.5% + JA-Me 0.5% was applied in the middle of internode segments, the secondary abscission zone was formed about 10 mm to 12 mm above to the acropetal direction of the treatment, as shown in Figure 6B. JA-Me interacts in some way with endogenous levels of IAA on the distance transport in the stem, and an auxin gradient between the end of JA-Me transport and the stem unaffected by JA-Me induces the secondary abscission zone. It seems that the most important factor for the induction of the secondary abscission zones in the internode of *B*. *calycinum* is the qualitative and quantitative balance between IAA and JA-Me. As mentioned above, an auxin gradient spanning the abscission zone has been proposed to regulate the timing of organ separation [42]. The final process of the secondary abscission zone is similar to that of the primary abscission zone, and both processes are connected with auxin gradient, as shown in Figure S3.

Histological and ultrastructural studies of McManus et al. [28] and Osborne [1] on bean explants indicated that the primary and the secondary abscissions were essentially the same during transdifferentiation processes occurring in cell separation zones. In contrast, in many species, including results of this study in *B. calycinum,* there are anatomical evidences that differentiation of the abscission zone is accompanied by cell divisions. For instance, cell divisions were involved in differentiation of the secondary abscission zone in the stem of excised gibberellin-treated cotyledonary nodes of cotton [47] and *Phaseolus vulgaris* [19]. Cell division is also associated with flower abscission induced by ABA and ethylene treatment of *Lupinus luteus* [48]. As shown in Figure 2 and Figure 3, the abscission zone was characterized by the presence of newly synthesized cell plates that resulted from periclinal cell division within one layer of mother cells in the stems of *B. calycinum*. In our study, anatomical examination of the secondary abscission in *B. calycinum* revealed that the effects of JA-Me 0.5% and IAA 0.1% on the formation of the separation layer were quite similar.

Cadmium accelerated premature senescence and leaf abscission in beans (*Phaseolus vulgaris*). Cd induced the formation of a secondary abscission zone, mainly at the secondary pulvinus, and the abscission was always initiated in epidermal and outer cortical cells of the pulvinus, thus the petiole remains attached to the stem [49]. These authors suggest that cell division and cell differentiation occur prior to the Cd-induced abscission in essentially the same way as in Cd-free bean plants documented by Webster [50,51] but the site of abscission zone development was clearly different, and the mechanism of the induction is unknown.

Maksymiec et al. [52] showed that Cd and Cu stimulated jasmonates accumulation in whole *Arabidopsis thaliana* and *Phaseolus coccineus* plants, and recently Alikhani and Abbaspour [53] documented that Cd induced an essential step of jasmonic acid biosynthesis, allene oxide cyclase (AOC) gene expression in wheat seedlings. Thus, it is possible that the Cd-induced secondary abscission zone in *Phaseolus vulgaris* is going through stimulation of jasmonates biosynthesis by Cd.

It is well known that some JAs-induced physiological and biochemical processes in plants are reduced by auxins, and some auxin-stimulated processes are inhibited by JAs [54,55]. Evidence for a close functional relationship between JAs signaling pathway and auxin homeostasis has been documented [56,57,58,59,60]. Hvoslef-Eide et al. [61] compared genes involved in the secondary abscission induced in *Euphorbia pulcherrima* (poinsettia) and in the primary abscission in *Pisum sativum* (pea). They documented that both species share at least six genes involved in the secondary and the primary abscission and indicated a high similarity between these processes. The differentiation of the style abscission zone (secondary abscission) in citrus is connected with an increase in expression of four transcription factors, one of them being peptide hormone ligand (CitIDA) [62]. The peptide INFLORESCENCE DEFICIENT IN ABSCISSION (IDA) controls abscission in *Arabidopsis* and *Citrus* [63]. Further studies on the effect of JA-Me and/or IAA on the expression of these genes will be required to explain the mode of actions of these compounds to induce the secondary abscission in *B. calycinum* in molecular levels.

## 4. Materials and Methods

### 4.1. Plant Materials and Hormone Treatment

Two to six-month-old plants of *Bryophyllum calycinum* Salisb. (Crassulaceae), propagated from epiphyllous buds arising in the marginal notches of the leaves, were used in the experiments. Different types of stem segments and decapitated stems of growing plants were used for the treatments of indole-3-acetic acid (IAA) at a concentration of 0.1% and 0.5%, methyl jasmonate (JA-Me) at a concentration of 0.5%, and a mixture of these hormones. All treatments of JA-Me 0.5%, IAA 0.1% and 0.5%, JA-Me 0.5% + IAA 0.1% (*w*/*w*) in lanolin paste or lanolin only were applied as a 2–3 mm stripe on the middle part or upper part around the internode or on top of the internode. For the experiments, mostly the second or third internodes from the top of growing plants with active elongation were used. Treatments with IAA and JA-Me were as follows:

**Experiment 1**: The effect of IAA at 0.1% on the secondary abscission zone formation in the excised internode segments without nodes and with lower nodes at a length of about 4 cm when IAA was applied to the middle of the internode of *B. calycinum* was investigated. The internode segments treated with lanolin only were the control segments. Segments were kept in the normal (natural) and inverted positions in a 50 mL glass chamber with water or water-moistened papers at the base of these segments, and under natural light conditions in a greenhouse. Experiments were repeated at least five times with 10 to 15 segments. 

A similar experiment concerning the formation of the secondary abscission zone was made on intact growing plants. After decapitation of the apical part of the shoot, IAA was applied to the middle of the last internode. Experiments were repeated at least seven times with ten or more growing plants, as shown in Figure S1. 

The plant materials shown in Figure 1A,B were used for histological investigations related to the formation of the secondary abscission zone, and were subjected to physicochemical investigations of auxin.

**Experiment 2:** The effects of IAA at 0.5% and JA-Me at 0.5%, their simultaneous application, and the difference in the place of the treatment on the formation of the secondary abscission zone in the excised segments of internode at 5 cm to 6 cm length kept in the normal (natural) and inverted positions were investigated. Experiments were repeated four times with ten or more segments. The experimental design and the places of the treatment of IAA, JA-Me and their mixture are shown in Figure 6. Others are the same as Experiment 1. The plant materials shown in Figure 6A were used for histological studies related to the formation of the secondary abscission zone induced by JA-Me.

**Experiment 3:** The effects of IAA 0.5% and JA-Me 0.5%, their simultaneous application, and the difference in the place of the treatment on the formation of the secondary abscission zone in the excised stem segments with nodes below and above the internode at 6 cm to 7 cm length kept in the normal and inverted positions were investigated. Experiments were repeated four times with ten or more segments. Experimental design and the place of the treatments of IAA, JA-Me and their mixture are shown in Figure S2. Others are the same as **Experiment 1**.

### 4.2. Histological Observations 

For histological observation, 0.7 cm pieces of stem in the middle of the internodes were cut 1 cm above the point of the application of the lanolin paste of IAA at 0.1% and JA-Me at 0.5% on the sixth, the eighth and the ninth day after treatment. Five samples of shoots were collected for each treatment. Pieces of the stem internode treated with lanolin only, IAA at 0.1% and JA-Me at 0.5% were collected. The materials were fixed in a chromic acid, acetic acid and formalin (CrAF) solution for 48 h at room temperature, dehydrated through an increasing alcohol series (70%, 80%, 90% and 100%), and embedded in paraffin. Longitudinal sections, 15 μm thick, were cut with a rotary microtome (Leica, Wetzelar, Germany) and stained with safranin (1% prepared in ultrapure water) followed by fast green (1% prepared in 95% ethanol). The sections were mounted in Canada balsam and analysed using a light microscope (Eclipse 80i, Nikon, Tokyo, Japan) with imaging software NIS-Elements BR ver. 4.00 (Nikon Instruments Inc., Tokyo, Japan) for photo documentation. 

### 4.3. Analyses of Plant Hormones in Relation to the Secondary Abscission Zone Formation Induced by IAA in the Stem Segments of B. calycinum

The stem segments of the experiment presented in Figure 1A,B were used for plant hormone analysis. The following samples of the stem were taken for analysis after treatment with IAA: the top yellowing part at the early stage of secondary abscission formation (zone 1), the green part above treatment (zone 2), and the green part of the internode below treatment (zone 3). Control samples were taken from equivalent zones in the segments treated with lanolin only. All plant material was collected at the same time, frozen in liquid N_2_ and lyophilized. Analyses of plant hormones were performed according to the methods reported previously [64,65,66]. Approximately 20 mg of powdered plant material was spiked with a mixture of stable isotope-labeled plant hormone, 20 pmol each, used as an internal standard. The samples were extracted with an organic solvent (methanol: water: formic acid = 15:4:1, *v*/*v*/*v*) three times. After evaporation under N_2_, the samples were resuspended in 3% methanol in 1 M formic acid, and then cleaned up on hybrid SPE cartridges (BondElut Plexa PCX, Agilent, Santa Clara, CA, USA). Qualitative and quantitative analyses of the plant hormones were performed in three replicates on a UHPLC-MS/MS system (Agilent Infinity 1260, Agilent, Waldbronn, Germany; coupled to a triple quadruple mass spectrometer MS/MS, 6410 Triple Quad LC/MS, Agilent, Santa Clara, CA, USA). Chromatographic separation was achieved on AscentisExpress RP-Amide analytical column (particle size 2.7 μm; 2.1 mm × 150 mm; Supelco, Bellefonte, PA, USA) in gradient mode. Analyses were carried out in positive electrospray ionization (ESI) mode using multiple reaction monitoring (MRM) transitions for the identification and quantification of all compounds of interest. Quantitation was based on calibration curves obtained with each pure standard compound, taking into account the recovery rates of the internal standard used. Technical details are given in the cited references.

### 4.4. Statistical Analysis

Three-way analysis of variance (ANOVA) was conducted using STATISTICA software (StatSoft Poland, Krakow, Poland). For a comparison of the means, post hoc differences were tested using Duncan’s multiple range test. *p* values of <0.05 were considered to be statistically significant. Values are expressed as the mean with standard error (*n* = 3). 

## 5. Conclusion

In the present study, we provided evidence that the position of the secondary abscission zone in the stem segments of *B. calycinum* can be manipulated by specific hormonal cues. The secondary abscission zone induced by 0.1% IAA is always formed at the site of the green-yellow tissue junction of the internode, and it is associated with relatively low levels of IAA in the yellowing top part of stem segments (above the abscission zone formation) in IAA-treated segments. JA-Me applied on its own to the middle of the internode substantially induced the formation of the secondary abscission zone, however, the simultaneous application with IAA at 0.5% substantially inhibited its formation in the basipetal direction. Thus, the secondary abscission zone induced by JA-Me might be cause by somehow decreasing auxin activity. Cell separation was associated with additional anticlinal cell division forming a periderm-like layer. The effects of JA-Me 0.5% and IAA 0.1% on the formation of the separation layer were quite similar.

## Supplementary Materials

Supplementary materials can be found at https://www.mdpi.com/1422-0067/21/8/2784/s1.

## References

[1] Osborne D.J., Roubelakis-Angelakis K.A., Thanh Van K.T. (1993). Morphogenetic signals and markers in vitro and in vivo. Morphogenesis in Plants. Molecular Approaches.

[2] Van Doorn W.G., Stead A.D. (1997). Abscission of flowers and floral parts. J. Exper. Bot..

[3] Roberts J.A., Whitelaw C.A., Gonzalez-Carranza Z.H., McManus M.T. (2000). Cell separation processes in plants—Models, mechanisms and manipulation. Ann. Bot..

[4] Taylor J.E., Whitelaw C.A. (2001). Signals in abscission. New Phytol..

[5] Tucker M.L., Kim J. (2015). Abscission research: What we know and what we still need to study. Stewart Postharvest Rev..

[6] Partharkar O.R., Walker J.C. (2018). Advances in abscission signaling. J. Exp. Bot..

[7] Bleecker A.B., Patterson S.E. (1997). Last exit: Senescence, abscission, and meristem arrest in Arabidopsis. Plant Cell.

[8] Roberts J.A., Elliott K.A., Gonzalez-Carranza Z.H. (2002). Abscission, dehiscence, and other cell separation processes. Annu. Rev. Plant Biol..

[9] Patterson S.E. (2001). Cutting loose. Abscission and dehiscence in Arabidopsis. Plant Physiol..

[10] Ito Y., Nakano T. (2015). Development and regulation of pedicel abscission in tomato. Front. Plant Sci..

[11] Meir S., Sundaresan S., Riov J., Agarwal I., Philosoph-Hadas S. (2015). Role of auxin depletion in abscission control. Stewart Postharvest Rev..

[12] Curtis R.W. (1984). Abscission-inducing properties of methyl jasmonate, ABA, and ABA-methylester and their interactions with ethephon, AgNO_3_ and malformin. J. Plant Growth Regul..

[13] Ueda J., Miyamoto K., Aoki M., Momotani Y., Kato J., Kamisaka S. The mode of actions of jasmonic acid and its methyl ester on the growth and the abscission. Proceedings of the 14th International Conference on Plant Growth Substances.

[14] Ueda J., Miyamoto K., Hashimoto M. (1996). Jasmonates promote abscission in bean petiole explants: Its relationship to the metabolism of cell wall polysaccharides and cellulase activity. J. Plant Growth Regul..

[15] Saniewski M., Węgrzynowicz E. (1995). Methyl jasmonate-induced leaf abscission in *Kalanchoe blossfeldiana*. Acta Hortic..

[16] Saniewski M., Gajewska E., Urbanek H. (1995). Activity of cell wall degrading glycanases in methyl jasmonate-induced leaf abscission in *Kalanchoe blossfeldiana*. Acta Agrobot..

[17] Hartmond U., Yuan R., Burns J.K., Grant A., Kender W.J. (2000). Citrus fruit abscission induced by methyl-jasmonate. J. Am. Soc. Hortic. Sci..

[18] Fidelibus M., Cathline K. (2010). Dose and time dependent effects of methyl jasmonate on abscission of grapes. Acta Hortic..

[19] Webster B.D., Leopold A.C. (1972). Stem abscission in *Phaseolus vulgaris* explants. Bot. Gaz..

[20] Pierik R.L.M. (1977). Induction of secondary abscission in apple pedicels in vitro. Physiol. Plant..

[21] Pierik R.L.M. (1980). Hormonal regulation of secondary abscission in pear pedicels in vitro. Physiol. Plant..

[22] Warren Wilson P.M., Warren Wilson J., Addicott F.T., McKenzie R.H. (1986). Induced abscission sites in internodal explants of *Impatiens sultani*: A new system for studying positional control. With an appendix: A mathematical model for abscission sites. Ann. Bot..

[23] Warren Wilson J., Warren Wilson P.M., Walker E.S. (1987). Abscission sites in nodal explants of *Impatiens sultani*. Ann. Bot..

[24] Warren Wilson J., Walker E.S., Warren Wilson P.M. (1988). The role of basipetal auxin transport in the positional control of abscission sites induced in *Impatiens sultani* stem explants. Ann. Bot..

[25] Warren Willson J., Palni L.M.S., Warren Wilson P.M. (1999). Auxin concentration in nodes and internodes of *Impatiens sultani*. Ann. Bot..

[26] Suzuki T. (1991). Shoot-tip abscission and adventitious abscission of internode in mulberry (*Morus alba*). Physiol. Plant..

[27] Plummer J.A., Vine J.H., Mullins M.G. (1991). Regulation of stem abscission and callus growth in shoot explants of sweet orange [*Citrus sinensis* (L.) Osbeck]. Ann. Bot..

[28] McManus M.T., Thompson D.S., Merriman C., Lyne L., Osborne D.J. (1998). Transdifferentiation of mature cortical cells to functional abscission cell in bean. Plant Physiol..

[29] Pang Y., Zhang J., Cao J., Yi S.-Y., He X.-Q., Cui K.-M. (2008). Phloem transdifferentiation from immature xylem cells during bark regeneration after girdling in *Eucommia ulmoides* Oliv. J. Exp. Bot..

[30] Yamaguchi M., Goué N., Igarashi H., Ohtani M., Nakano Y., Mortimer J.C., Nishikubo N., Kubo M., Katayama Y., Kakegawa K. (2010). VASCULAR-RELATED NAC-DOMAIN6 and VASCULAR-RELATED NAC-DOMAIN7 effectively induce transdifferentiation into xylem vessel elements under control of an induction system. Plant Physiol..

[31] Reusche M., Thole K., Janz D., Truskina J., Rindfleisch S., Drübert C., Polle A., Lipka V., Teichmann T. (2012). *Verticillium* infection triggers VASCULAR-RELATED NAC-DOMAIN7-dependent de novo xylem formation and enhances drought tolerance in *Arabidopsis*. Plant Cell.

[32] Saniewski M., Ueda J., Miyamoto K. (2000). Methyl jasmonate induces the formation of secondary abscission zone in stem of *Bryophyllum calycinum* Salisb. Acta Physiol. Plant..

[33] Saniewski M., Góraj-Koniarska J., Gabryszewska E., Miyamoto K., Ueda J. (2016). Auxin effectively induces the formation of the secondary abscission zone in *Bryophyllum calycinum* Salisb. (Crassulaceae). Acta Agrobot..

[34] Novák O., Hényková E., Sairanen I., Kowalczyk M., Pospišil T., Ljung K. (2012). Tissue-specific profiling of the *Arabidopsis thaliana* auxin metabolome. Plant J..

[35] Reinecke D.M., Bandurski R.S. (1981). Metabolic conversion of ^14^C-indole-3-acetic acid to ^14^C-oxindole-3-acetic acid. Biochem. Biophys. Res. Commun..

[36] Reinecke D.M., Bandurski R.S. (1983). Oxindole-3-acetic acid, an indole-3-acetic acid catabolite in *Zea mays*. Plant Physiol..

[37] Ludwig-Müller J. (2011). Auxin conjugates: Their role for plant development and in the evolution of land plants. J. Exp. Bot..

[38] Ljung K. (2013). Auxin metabolism and homeostasis during plant development. Development.

[39] Pěnčík A., Simonovik B., Petersson S.V., Henyková E., Simon S., Greenham K., Zhang Y., Kowalczyk M., Estelle M., Zažímalová E. (2013). Regulation of auxin homeostasis and gradients in *Arabidopsis* roots through the formation of the indole-3-acetic catabolite 2-oxindole-3-acetic acid. Plant Cell.

[40] Kramer E.M., Ackelsberg E.M. (2015). Auxin metabolism rates and implications for plant development. Front. Plant Sci..

[41] Addicott F.T. (1982). Abscission.

[42] Addicott F.T., Lynch R.S., Carns H.R. (1955). Auxin gradient theory of abscission regulation. Science.

[43] Jin X., Zimmermann J., Polle A., Fischer U. (2015). Auxin is a long-range signal that acts indenpendently of ethylene signaling on leaf abscission in Populus. Front. Plant Sci..

[44] Seo H.S., Song J.T., Cherong J.-J., Lee Y.-H., Lee Y.-W., Hwang I., Lee J.S., Chi Y.D. (2001). Jasmonic acid carboxyl methyltransferase: A key enzyme for jasmonatte-regulated plant responses. Proc. Natl. Acad. Sci. USA.

[45] Sato C., Aikawa K., Sugiyama S., Nabeta K., Masuta C., Matsuura H. (2011). Distal transport of exogenously applied jasmonyl-isoleucine with wounding stress. Plant Cell Physiol..

[46] Tomogami S., Noge K., Abe M., Agrawal G.K., Rakwal R. (2012). Methyl jasmonate is transported to distal leaves via vascular process metabolizing itself into JA-Ile and triggering VOCs emission as defensive metabolites. Plant Signal. Behav..

[47] Bornman C.H., Addicott F.T., Lyon J.L., Smith O.E. (1968). Anatomy of gibberellin-induced stem abscission in cotton. Am. J. Bot..

[48] Wilmowicz E., Kućko A., Ostrowski M., Panek K. (2018). Inflorescence deficient in abscission-like is an abscission-associated and phytohormone-regulated genes in flower separation of *Lupinus luteus*. Plant Growth Regul..

[49] Vazquez M.D., Poschenrieder C., Barcelo J. (1989). Pulvinus structure and leaf abscission in cadmium -treated bean plants (*Phaseolus vulgaris*). Can. J. Bot..

[50] Webster B.D. (1970). A morphogenetic study of leaf abscission in *Phaseolus vulgaris*. Am. J. Bot..

[51] Webster B.D. (1973). Ultrastructural studies of leaf abscission in *Phaseolus*: Ethylene effects on cell walls. Am. J. Bot..

[52] Maksymiec W., Wianowska D., Dawidowicz A.L., Radkiewicz S., Mardarowicz M., Krupa Z. (2005). The level of jasmonic acid in *Arabidopsis thaliana* and *Phaseolus coccineus* plants under heavy metal stress. J. Plant Physiol..

[53] Alikhani O., Abbaspour H. (2019). Effects of methyl jasmonate and cadmium on growth traits, cadmium transport and accumulation, and allene-oxide cyclase gene expression in wheat seedlings. Rev. Agric. Neotrop..

[54] Saniewski M., Ueda J., Miyamoto K. (2002). Relationship between jasmonates and auxin in regulation of some physiological processes in higher plants. Acta Physiol. Plant..

[55] Ishimaru Y., Hayashi K., Suzuki T., Fukai H., Prusinska J., Meester C., Quareshy S., Egoshi S., Matsuura H., Takahashi K. (2018). Jasmonic acid inhibits auxin-induced lateral rooting independently of the CORONATINE INSENSITIVE1 receptor. Plant Physiol..

[56] Hentrich M., Bottcher C., Duchting P., Cheng Y., Zhao Y., Berkowitz O., Masle J., Medina J., Pollmann S. (2013). The jasmonic acid signaling pathway is linked to auxin homeostasis through the modulation of YUCCA8 and YUCC9 gene expression. Plant J..

[57] Perez A.C., Goossens A. (2013). Jasmonate signaling a copycat of auxin signaling?. Plant Cell Environ..

[58] Du H., Liu H., Xiong L. (2013). Endogenous auxin and jasmonic acid levels are differentially modulated by abiotic stresses in rice. Front. Plant Sci..

[59] Pazmino D.M., Rodriguez-Serrano M., Romero-Puertas M.C., Sandalio L.M. (2014). Regulation of epinasty induced by 2,4-dichlorophenoxyaccetic acid in pea and *Arabidopsis* plants. Plant Biol..

[60] Gutierrez L., Mongelard G., Flokova K., Pacurar D.J., Novak O., Staswick P., Kowalczyk M., Pacurar B.M., Demailly H., Geiss G. (2012). Auxin control *Arabidopsis* adventitious root initiation by regulating jasmonic acid homeostasis. Plant Cell.

[61] Hvoslef-Eide A.K., Munster C.M., Mathiesen C.A., Ayeh K.O., Melby T.I., Rrasolomanana P., Lee Y.-K. (2016). Primary and secondary abscission in *Pisum sativum* and *Euphorbia pulcherrima*—How do they compare and how do they differ?. Front. Plant Sci..

[62] Estornell L.H., Gomez M.D., Perez-Amador M.A., Talon M., Tadeo F.R. (2016). Secondary abscission zones: Understanding the molecular mechanisms triggering stylar abscission in citrus. Acta Hortic..

[63] Estornell L.H., Weioldhagen M., Perez-Amador M.A., Talon M., Tadeo F.R., Butenko M.A. (2015). The IDA peptide controls abscission in Arabidopsis and Citrus. Front. Plant Sci..

[64] Dziurka M., Janeczko A., Gullner G., Oklestková J., Novák O., Saja D., Skoczowski A., Tóbiász J., Barna B. (2016). Local and systemic hormonal responses in pepper leaves during compatible and incompatible pepper-tobamovirus interactions. Plant Physiol. Biochem..

[65] Płażek A., Dubert F., Kopeć P., Dziurka M., Kalandyk A., Pastuszak J., Wolko B. (2018). Seed hydropriming and smoke water significantly improve low-temperature germination of *Lupinus angustifolius* L.. Int. J. Mol. Sci..

[66] Wiszniewska A., Koźmińska A., Hanus-Fajerska E., Dziurka K. (2019). Insight into mechanism of multiple stresses tolerance in a halophyte Aster tripolium subjected to salinity and heavy metal stress. Ecotox. Environ. Safety.

