# Supplementary material for: Formation of the Secondary Abscission Zone Induced by the Interaction of Methyl Jasmonate and Auxin in Bryophyllum calycinum: Relevance to Auxin Status and Histology
Source: Int J Mol Sci. 2020 Apr 16;21(8):2784. doi: 10.3390/ijms21082784 (PMC7215696; doi:10.3390/ijms21082784)
Supplement: Supplementary file 1 [file ijms-21-02784-s001.pdf]

Supplementary Material

Table S1. Effect of IAA at a concentration of 0.1% applied in the middle of the internode segments of *B. calycinum* on endogenous levels of some auxins.

Auxins, pmol/gDW	Top		Above Treatment		Below Treatment	
	Control	IAA 0.1%	Control	IAA 0.1%	Control	IAA 0.1%
IAA	1.283 ± 0.117b	1.168 ± 0.071b	0.535 ± 0.056a	4.557 ± 0.096d	0.298 ± 0.029 a	3.237 ± 0.064c
IAAsp	0.144 ± 0.004c	0.090 ± 0.001b	0.083 ± 0.001ab	0.16 4 ± 0.001d	0.071 ± 0.003a	0.342 ± 0.013e
IAA-Glut	0.251 ± 0.007b	0.239 ± 0.002b	0.244 ± 0.004b	0.271 ± 0.011c	0.206 ± 0.005a	0.241 ± 0.003b
IAA-Me	0.096 ± 0.011b	0.198 ± 0.004c	0.100 ± 0.002 b	0.099 ± 0.005b	0.077 ± 0.001 a	0.103 ± 0.009b
IAA-carb	1.065 ± 0.142ab	3.261 ± 0.628c	1.800 ± 0.202 b	7.839 ± 0.367 d	0.551 ± 0.121a	7.562 ± 0.535d
Ox-IAA	0.847 ± 0.052ab	1.901 ± 0.048b	0.106 ± 0.009 a	37.220 ± 0.158c	0.024 ± 0.004a	43.344 ± 1.078d
4-Cl-IAA	0.074 ± 0.008ab	0.318 ± 0.020d	0.073 ± 0.005ab	0.191 ± 0.019c	0.054 ± 0.005 a	0.108 ± 0.010 b
5-Cl-IAA	4.237 ± 0.124d	2.777 ± 0.156bc	2.534 ± 0.106b	4.582 ± 0.177d	1.710 ± 0.177a	3.072 ± 0.128c
IBA	0.45 ± 0.08a	0.58 ± 0.06b	0.56 ± 0.10ab	0.63 ± 0.05 b	0.53 ± 0.10 ab	0.55 ± 0.01 ab

IAA: indole-3-acetic acid; IAAsp: indole-3-acetyl-L-aspartic acid; IAA-Glut: indole-3-acetyl-4-glutamic acid; IAA-Me: indole-3-acetic acid methyl ester; IAA-carb: indole-3-carboxylic acid; OxIAA: oxindole-3-acetic acid; 4-Cl-IAA: 4-chloroindole-3-acetic acid; 5-Cl-IAA: 5-chloroindole-3-acetic acid; IBA: indole-3-butyric acid. Values are expressed as the mean ± SE ($n = 3$); Different letters indicate statistic difference by Duncan's Multiple Range Test, with $p < 0.05$ after ANOVA.

Table S2. Effect of IAA at a concentration of 0.1% applied in the middle of the internode segments of *B. calycinum* on endogenous levels of abscisic acid (ABA), salicylic acid (SA), benzoic acid, (BA), JA (jasmonic acid), JA-Me and 12-oxo-phytodienoic acid (OPDA).

Compound, pmol/gDW	Top		Above Treatment		Below Treatment	
	Control	IAA 0.1%	Control	IAA 0.1%	Control	IAA 0.1%
ABA	0.289 ± 0.017a	0.633 ± 0.015c	0.271 ± 0.022a	0.525 ± 0.062b	0.254 ± 0.011a	0.472 ± 0.034b
SA	17.19 ± 0.64c	10.31 ± 0.36a	8.78 ± 0.270a	10.74 ± 0.95a	13.19 ± 0.96b	9.73 ± 1.15a
BA	6.11 ± 0.43d	4.11 ± 0.05a	5.18 ± 0.15bc	4.72 ± 0.70ab	5.74 ± 0.15cd	4.08 ± 0.20a
JA	5.39 ± 0.05a	9.43 ± 0.33b	17.38 ± 1.11c	8.49 ± 0.14ab	22.47 ± 2.04d	7.87 ± 0.51ab
JA-Me	0.083 ± 0.003a	0.091 ± 0.002abc	0.093 ± 0.002bc	0.099 ± 0.004c	0.083 ± 0.003a	0.088 ± 0.004ab
OPDA	0.247 ± 0.011ab	0.244 ± 0.011ab	0.274 ± 0.004b	0.317 ± 0.011c	0.229 ± 0.008a	0.264 ± 0.014a

ABA: abscisic acid; SA: salicylic acid; BA: benzoic acid; JA: jasmonic acid; JA-Me: jasmonic acid methyl ester; OPDA: 12-oxo-phytodienoic acid;.Values are expressed as the mean ± SE ($n = 3$); Different letters indicate statistic difference by Duncan's Multiple Range Test, with $p < 0.05$ after ANOVA.

Table S3. Effect of IAA at a concentration of 0.1% applied in the middle of the internode segments of *B. calycinum* on endogenous levels of some cytokinins.

Cytokinins, fmol/gDW	Top		Above Treatment		Below Treatment	
	**Control**	**IAA 0.1%**	**Control**	**IAA 0.1%**	**Control**	**IAA 0.1%**
t-Z	28.5 ± 1.7ab	20.4 ± 5.2a	40.9 ± 5.2bc	32.1 ± 6.9abc	46.1 ± 6.8c	73.1 ± 2.8d
c-Z	27.1 ± 1.9ab	23.3 ± 1.6a	32.0 ± 2.4bc	30.4 ± 3.5ab	30.8 ± 1.0ab	39.7 ± 3.4c
t-ZR	158.5 ± 5.2e	41.2 ± 6.2b	104.8 ± 1.6d	31.8 ± 1.5ab	76.4 ± 1.5c	27.0 ± 0.5a
c-ZR	22.5 ± 0.7b	20.6 ± 0.7ab	20.9 ± 0.3ab	22.1 ± 0.5ab	20.7 ± 3.0ab	18.0 ± 0.2a
IP	90.2 ± 1.6b	80.8 ± 3.7a	92.8 ± 3.3b	94.4 ± 3.3b	86.7 ± 2.6ab	87.1 ± 1.0ab
IPAD	34.3 ± 0.4d	6.6 ± 1.1a	21.3 ± 0.5c	6.1 ± 0.6a	14.5 ± 0.3b	5.5 ± 0.1a
KIN	31.2 ± 3.8ab	27.6 ± 3.3a	43.8 ± 4.8c	41.2 ± 1.2bc	37.3 ± 3.0abc	29.7 ± 2.0a
KIN-R	8.9 ± 1.3a	7.0 ± 0.3a	8.5 ± 0.0a	8.0 ± 0.4a	6.9 ± 0.5a	8.1 ± 0.5a

t-Z: *trans*-zeatin; c-Z: *cis*-zeatin; t-ZR: *trans*-zeatin-7-riboside; c-ZR: *cis*-zeatin-riboside; IP: isopentenyladenine; IPAD: isopentenyladenosine; KIN: kinetin; KIN-R kinetin riboside; Values are expressed as the mean ± SE (n = 3); Different letters indicate statistic difference by Duncan's Multiple Range Test, with $p < 0.05$ after ANOVA.

Table S4. Effect of IAA at a concentration of 0.1% applied in the middle of the internode segments of *B. calycinum* on endogenous levels of some gibberellins.

GAs, pmol/gDW	Top		Above Treatment		Below Treatment	
	**Control**	**IAA 0.1%**	**Control**	**IAA 0.1%**	**Control**	**IAA 0.1%**
GA_1	0.42 ± 0.06a	0.56 ± 0.09a	0.58 ± 0.11a	0.53 ±0.09a	0.46 ± 0.05	0.49 ± 0.03a
GA_3	1.45 ± 0.21bc	1.19 ±0. 19ab	2.32 ± 0.26d	2.00 ± 0.17cd	2.01 ± 0.22cd	0.62 ± 0.10a
GA_4	0.07 ± 0.00c	0.03 ± 0.01a	0.04 ±0.01ab	0.05 ±0.01ab	0.04 ± 0.01ab	0.05 ± 0.01bc
GA_5	0.26 ± 0.02a	0.22 ± 0.03a	0.21 ± 0.02a	0.69 ± 0.05c	0.14 ± 0.02a	0.55 ± 0.08b
GA_6	0.83± 0.06a	0.90 ± 0.03ab	0.91 ±0.01ab	0.89 ± 0.02ab	0.82 ± 0.05a	1.02 ± 0.09b
GA_7	0.24 ± 0.02b	0.15 ± 0.02a	0.13 ± 0.02a	0.21 ± 0.04ab	0.21 ±0.01ab	0.17 ± 0.03ab
GA_9	0.20 ± 0.03c	0.05 ± 0.01ab	0.07 ± 0.01ab	0.07 ± 0.01ab	0.04± 0.05a	0.10 ± 0.02b
GA_{20}	0.14± 0.01a	0.28 ± 0.01bc	0.15 ± 0.01ab	0.37± 0.06c	0.09± 0.01a	0.34 ±0.09c

Values are expressed as the mean ± SE (n = 3); Different letters indicate statistic difference by Duncan's Multiple Range Test, with $p < 0.05$ after ANOVA.

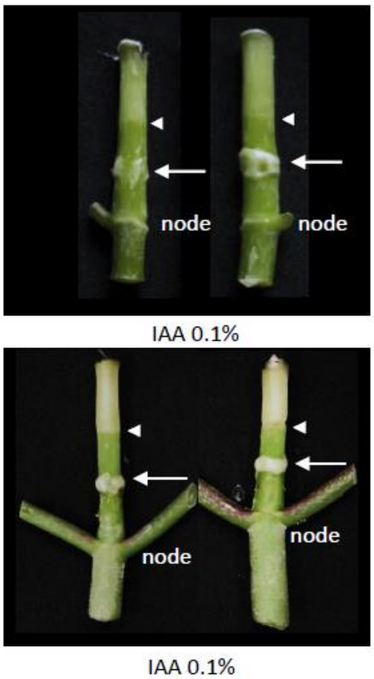

Figure S1. The effect of IAA at a concentration of 0.1% on the secondary abscission zone formation in the internode segments with lower node and in decapitated stem of *B. calycinum*. Upper figure: the secondary abscission zone formation in excised internode segment with lower node treated in the middle of the internode with IAA, photographed 9 days after treatment; lower figure: the secondary abscission zone formation in the last internode treated in the middle with IAA after decapitation of apical part of shoot in growing plant. White arrows indicate the place of treatment, and white arrowheads indicate the place of the formation of the secondary abscission zone. Photographs were taken 9 days after treatment.

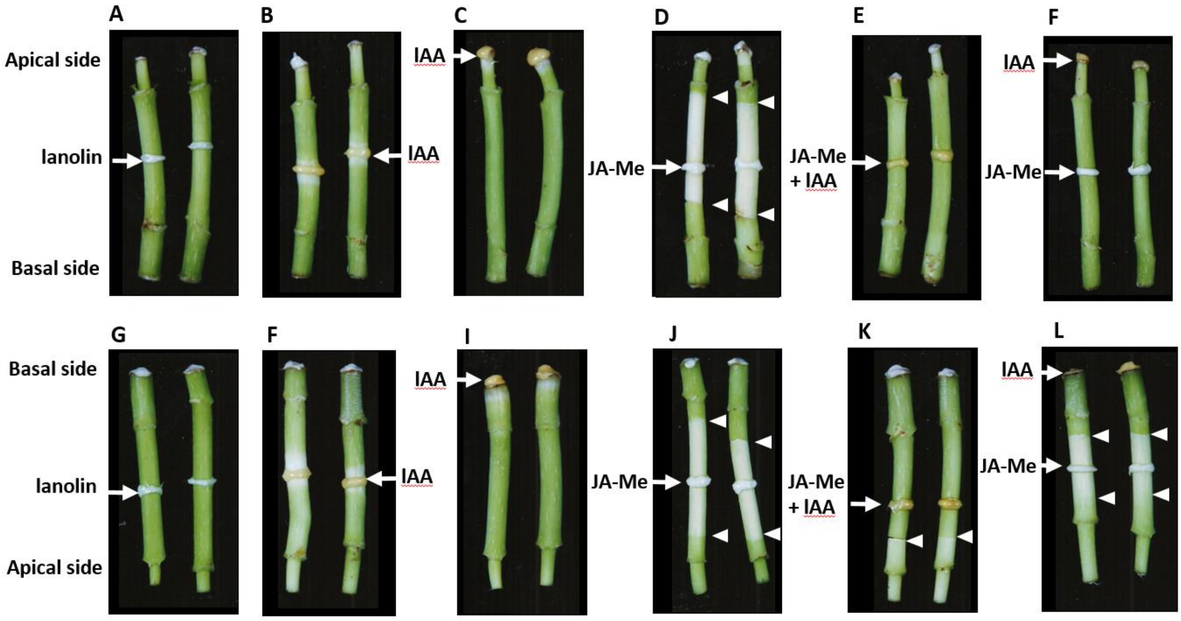

Figure S2. The effect of IAA 0.5% and/or JA-Me 0.5% on the formation of the secondary abscission zone depending on place of treatment in excised stem segments with nodes below and above the internode in *B. calycinum*. After the treatment, the excised segments were kept in normal (**A**–**F**) and inverted position (**G**–**L**), and then photographed 10 days after treatments. (**A**,**G**) control, lanolin applied on the top and in the middle of internode; (**B**,**H**) IAA alone applied in the middle of internode and lanolin on the top of internode; (**C**,**I**) IAA alone applied on the top of internode; (**D**,**J**) JA-Me alone

applied in the middle of internode and lanolin on the top; (**E**,**K**) JA-Me and IAA applied together in the middle of internode; (**F**,**L**) JA-Me applied in the middle and IAA applied on the top. White arrows indicate the place of treatment, and white arrowheads indicate the place of the formation of the secondary abscission zone.

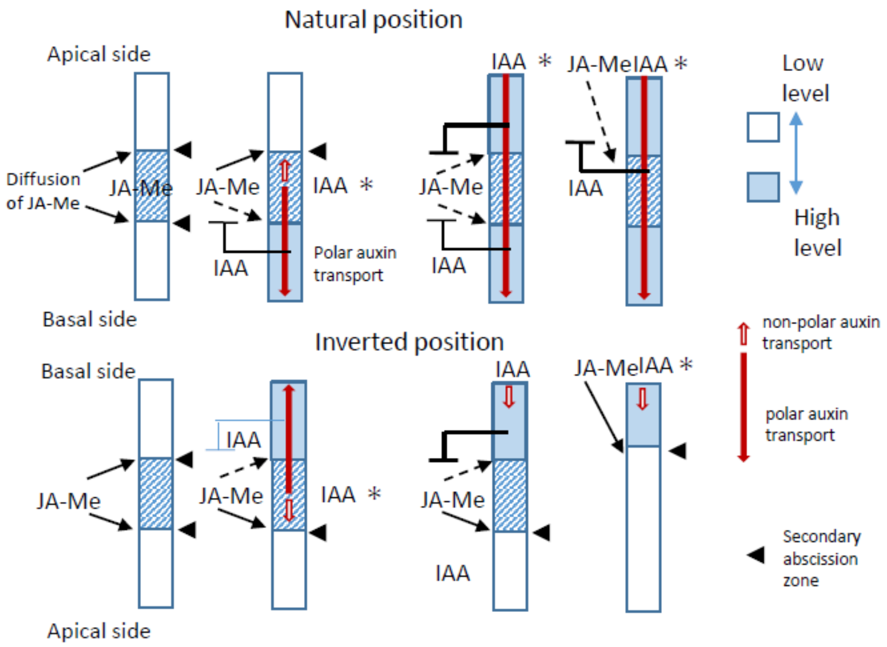

Figure S3. Diagrammatic representation of IAA and JA-Me interaction on the secondary abscission zone formation in the internode segments of *B. calycinum*.
